# Introducing Newborn Screening for Severe Combined Immunodeficiency—The New Zealand Experience

**DOI:** 10.3390/ijns8020033

**Published:** 2022-05-10

**Authors:** Natasha Heather, Mark de Hora, Shannon Brothers, Pippa Grainger, Detlef Knoll, Dianne Webster

**Affiliations:** 1 Newborn Metabolic Screening Programme, LabPlus, Auckland District Health Board, Auckland 1148, New Zealand; nheather@adhb.govt.nz (N.H.); mdehora@adhb.govt.nz (M.d.H.); shannonb@adhb.govt.nz (S.B.); detlefk@adhb.govt.nz (D.K.); 2 Liggins Institute, University of Auckland, Auckland 1142, New Zealand; 3 Starship Children’s Hospital, Auckland District Health Board, Auckland 1148, New Zealand; 4 Diagnostic Genetics, LabPlus, Auckland District Health Board, Auckland 1148, New Zealand; pgrainger@adhb.govt.nz

**Keywords:** severe combined immunodeficiency, T-cell receptor excision circles, newborn screening, neonatal screening, screening evaluation, screening terminology, screening target, case definitions

## Abstract

Screening for severe combined immunodeficiency (SCID) was added to the New Zealand national newborn screening programme in December 2017. Documentation pertaining to the application to add SCID to the panel and screening results over the first three years were reviewed. Screening evaluation metrics were shown to differ according to site of collection (babies in a neonatal intensive care unit vs. the community), definition of a positive test (out-of-range result vs. result leading to a further action on baby), and screening target/case definition (primary SCID vs. non-SCID T-cell lymphopenia). Our experience demonstrates both the value of close clinical involvement during the implementation phase of SCID screening and that the use of standard definitions will facilitate international comparison.

## 1. Introduction

Severe combined immune deficiency (SCID) is a profound inborn error of immunity which is uniformly fatal if not diagnosed and treated within the first months of life. Screening is based on the quantification of T-cell receptor excision circles (TREC), which are absent in babies who lack T cells. SCID newborn screening was first implemented in the North American State of Wisconsin, added to the Recommended Uniform Screening Panel (RUSP) in 2010, and adopted more slowly world-wide.

The New Zealand newborn bloodspot screening programme (known as the newborn metabolic screening programme) is fully government funded by the Ministry of Health National Screening Unit (NSU). Testing and follow-up services are delivered from a single laboratory in Auckland (LabPlus, Auckland City Hospital, Auckland, New Zealand). About 99.5% of the approximately 60,000 births annually are screened for amino acid breakdown disorders, biotinidase deficiency, congenital adrenal hyperplasia, congenital hypothyroidism, cystic fibrosis, fatty acid oxidation disorders, galactosemia, and the most recently added disorder, severe combined immunodeficiency (SCID).

Programme governance is by the NSU under the guidance of a Technical Working Group which provides specialist paediatric input (endocrinology, biochemical genetics, immunology, and pulmonology), public health expertise, specialist midwifery, chemical pathology, and cultural support to the screening unit staff. Programme policy is well defined and publicly available and contains a pathway for adding disorders to the programme (and for removing disorders) [1,2].

The application to add SCID to the screening panel was made by the national paediatric immunology service and received by the NSU in 2013—the same year the Technical Working Group approved the application and the NSU proceeded to commission a cost-utility study. The study showed that the cost of screening per Quality Adjusted Life Year (NZD 30,000) was similar to that for other healthcare interventions [3], and accordingly, in 2015, the Technical Working Group and the New Zealand National Screening Committee approved the NSU to proceed to a budget bid for government funding. This was successful and screening commenced in December 2017.

The aim of this report is to document the initial New Zealand experience and SCID screening performance over the first three years. 

## 2. Materials and Methods

In New Zealand, routine NBS consists of a single heel-prick sample collected onto specialised collection paper within a recommended age of 48–72 h. Babies born ≤ 1500 g have a further routine sample collected at 2 weeks and those ≤1000 g another at one month, because of the known association of false negative newborn screening for congenital hypothyroidism when TSH is the primary screen. 

The laboratory method utilises the EnLite™ Neonatal TREC kit (Perkin Elmer, Turku, Finland). The method is a multiplex endpoint polymerase chain reaction (PCR) method that amplifies and quantifies the TREC DNA, with amplification of the β-actin gene as an internal control. The screening TREC cut-off was set at ≤18 copies/µL blood, with urgent paediatric notification at ≤5 copies/µL.

SCID screening was reviewed over the initial three-year period, from December 2017 to December 2020.

## 3. Results

### 3.1. Live Testing

We did not commence with a pilot programme as we considered there were no significant questions about the addition of SCID to the existing test panel. However, we considered the initial period one of live testing, where screening was closely monitored. At screening outset, the laboratory team met with a paediatric immunologist weekly to discuss assay results and action on positive tests (with the proviso that they would have been called immediately with a highly suspicious result). These meetings became less frequent as the screening and follow-up processes were tested. 

Initially, all results were referred for diagnostic testing, but the number of positive screens was higher than predicted so, following weekly discussion, the protocol was revised so that borderline positive results had a second screening sample requested and were only referred for diagnostic testing if the TREC remained below cut-off. 

SCID diagnostic samples included lymphocyte subsets and naïve T cell subset evaluation, obtained by minimally invasive heel stick collection. The flow cytometry assay was developed and optimised for the small volume, with analysis on a single platform in a single central testing laboratory. Challenges of timely delivery of appropriate diagnostic samples over a small but geographically diverse population were noted and addressed by the development of a national dedicated prefilled request form, which detailed sample requirements and the need for an urgent direct courier to the central laboratory. 

### 3.2. Screening Performance 

From 1 December 2017 to 30 November 2020, 191,075 babies were screened for SCID. A total of 101 babies had an out-of-range result, i.e., ≤18 copies, and 2 were diagnosed with primary SCID [4]. The 2 cases of primary SCID were urgently notified to the clinical immunology service aged 4 and 8 days and had diagnostic flow cytometry results available aged 7 and 15 days, respectively.

Out-of-range results by place of collection are shown in Table 1. Samples collected from babies in a neonatal unit (NICU) are from both pre-term infants requiring care for immaturity and term babies who are unwell. The definition of a positive screen varies between publications. We define a positive screen as a result which requires a further action on the baby (Table 1). Hence, a referral or a request for a second sample is a positive screen. An out-of-range result, when a further sample is scheduled to be taken, is not counted as a positive screen (although these results are reported with a reminder to collect the next scheduled sample). Similarly, an out-of-range SCID result when there has been a previous normal result does not trigger further action and is not a positive screen result. In total, 34/101 out-of-range SCID results were normal on a previous or subsequent scheduled sample (Table 2).

As reported by other newborn screening programmes, in addition to 2 cases of primary SCID, 2 with T-cell lymphopaenia due to pre-term/low birth weight alone, and 19 other babies with non-SCID T-cell lymphopaenia (TCL) were identified [4]. Non-SCID TCL diagnoses included 11 cases of syndromes with T-cell impairment with 2 congenital athymia (1 complete DiGeorge syndrome, 1 CHARGE association), 7 partial DiGeorge syndrome, 2 other, 7 cases of reversible conditions with T-cell impairment, and 1 idiopathic TCL. 

To date, no cases of SCID have been diagnosed which were not detected by the screening programme. New Zealand has a national paediatric immunology service, and such diagnoses would likely be reported to the screening programme. Screening sensitivity was 100% in this limited study.

**Figure 1:**
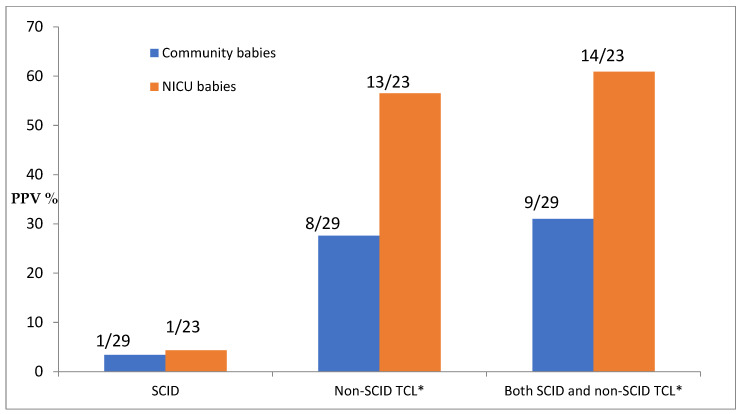
Positive predictive value (PPV) of screening by diagnosis and population. * TCL = T-cell lymphopenia; count here includes TCL due to preterm alone.

The positive predictive value of screening is dependent on both what is considered a positive test and what is considered the screened population, as shown in Figure 1.

Screening specificity is again dependent on what is counted—this was 99.95% when calculated for out-of-range results, but 99.97% for positive tests as defined by further action on the baby and SCID false positive tests including those with other TCL.

## 4. Discussion

The New Zealand newborn screening programme has a well-defined and documented pathway for adding new disorders, and this was successfully followed, leading to the addition of SCID to the screening panel in December 2017. Our NZ newborn screening results align with the expected population frequency of SCID of 1:50–100,000 [5]. Close clinical involvement has been critical to the success of both the application and implementation processes. Of particular value has been the availability of a paediatric immunologist to review screening results, quality parameters, and the screening algorithm. Paediatric immunology also provides short- and long-term follow-up for the programme.

Although the screening laboratory is well staffed and experienced, dealing with the molecular technology and a test with non-linear results proved challenging. Appropriate laboratory spaces for separate reagent preparation, DNA extraction, amplification and detection needed to be identified with workflow arrangements discussed and agreed with the diagnostic genetics department. Sufficient thermal cyclers were needed to ensure that turnaround times were achievable due to the length required to process each micro titre plate of samples (approximately 4 h). The external quality control scheme provided by the Centre for Disease Control and Prevention (Atlanta, GA, USA) has been important to ensuring the quality of testing has been appropriate. 

We did not consider a pilot programme as we considered there were no significant questions about the addition of SCID to the existing test panel. However, our experience of adding new disorders is that it is important to be prepared for unanticipated issues. We considered the initial period one of live testing, during which we identified a higher than anticipated rate of referral for diagnostic testing, and challenges in the delivery of diagnostic samples. These issues were able to be rapidly identified and resolved through the process of regular result review meetings scheduled between the laboratory and clinical immunology teams.

Screening metrics can be improved by the addition of second-tier genetic testing [6], although the screening laboratory would need to consider implementation regarding timeliness of result availability and management of updated sequence interpretation.

It is recognised that SCID screening in small and sick babies is different from that in term infants [7]. Our metrics similarly demonstrate this, with a far greater rate (a thirty-five-fold increase) in out-of-range results observed amongst samples collected from babies in NICUs as compared with those in the community. For valid comparability of screening parameters this group should be specified (e.g., by number) or analysed separately.

The difficulty of comparing screening metrics between programmes who use a common term with many different meanings is not new. A lexicon of screening terms was published by the International Society for Neonatal Screening in 2005 [8] with minimal impact. The newborn screening expert panel of the Clinical and Laboratory Standards Institute (CLSI) has published a glossary of terms [9], although this is incomplete at the time of writing. Further definitions particular to SCID screening will be included in the next edition of CLSI Newborn Screening for SCID guideline (NBS06) [10]. 

Blom et al. studied the problem and recently published recommendations for uniform definitions for use in SCID screening [11]. They recommend the use of normal value and abnormal value to distinguish between positive and negative screen results. We prefer the definition of a positive screen being a result which triggers an additional action on the baby. The terms are synonymous in the context of healthy community babies, but not in premature and sick babies who have additional routine screening samples scheduled. For example, the newborn screen on a 900 g birthweight baby consists of three tests done at 48 h, 2 weeks, and 4 weeks—the result of the screen takes into account the results of the three tests; thus, a low TREC abnormal value on the second or third sample when the first sample had a normal level would not be considered a positive screen, nor would a low value on the 48 h sample (unless the level was so low as to merit immediate paediatric referral). Further counting of abnormal results could mean that a baby had more than one positive screen which would confound determination of positive predictive value and sensitivity. 

We agree with Blom et al. that a clear definition of screening targets is important in defining sensitivity and positive predictive value [11]. Many paediatric immunologists have expressed the view that all clinically significant (actionable) lymphopenias should be the target of screening. However, the classification of conditions as primary (e.g., SCID) and secondary targets (e.g., other actionable lymphopenias) does not add clarity to the programme evaluation. It may be appropriate, at least in some contexts, to reframe the screening as for significant lymphopenias or similar terminology. We have chosen to provide separate metrics for SCID alone and together with other conditions. 

In summary, we consider SCID screening to have been successfully added to the New Zealand newborn screening panel but await further reassurance based on international comparisons with standard definitions.

## Figures and Tables

**Table 1:** SCID screening results by site of collection.

	*n*	Out-of-Range Results	Positive Tests *
Community collection	169,771	29 (0.02%)	29 (0.02%)
NICU collection	10,652	72 (0.7%)	27 (0.3%)

* Positive test defined as a test result which requires further action on a baby.

**Table 2:** Follow-up in NICU and community babies.

	NICU Babies, ≤1500 g Birthweight	NICU Babies, >1500 g Birthweight	Community Babies
*N*	1638	9014	169,771
*N* out-of-range results	48	24	29
*N* resolved on scheduled sample	34	na	na
Died (non-SCID)	10	1	0
*N* referrals *	4	16	15
Outcome	1 SCID 0 non-SCID TCL 2 TCL due to preterm alone	0 SCID 11 non-SCID TCL	1 SCID 8 non-SCID TCL

TCL = T-cell lymphopaenia; na = not applicable; * referrals = N referred for flow cytometry and excludes positive screening results that were resolved with a normal requested second sample.

## Data Availability

The data presented in this study are available on request from the corresponding author through application to the National Screening Unit, Ministry of Health.
